# In Vivo optical coherence tomography visualization of intraplaque neovascularization at the site of coronary vasospasm: a case report

**DOI:** 10.1186/s12872-016-0408-y

**Published:** 2016-11-25

**Authors:** Kenichi Tsujita, Koichi Kaikita, Satoshi Araki, Toshihiro Yamada, Suguru Nagamatsu, Kenshi Yamanaga, Kenji Sakamoto, Sunao Kojima, Seiji Hokimoto, Hisao Ogawa

**Affiliations:** 1 Department of Cardiovascular Medicine, Graduate School of Medical Sciences, Kumamoto University, 1-1-1 Honjo, Chuo-ku, Kumamoto 860-8556 Japan; 2 Department of Cardiovascular Medicine, National Cerebral and Cardiovascular Center, Suita, Japan

**Keywords:** Vasospasm, Neovascularization, Optical coherence tomography

## Abstract

**Background:**

Coronary plaques in patients with coronary vasospastic angina have been characterized by diffuse intima-media thickening with homogeneous fibrous tissue, without confluent necrotic tissue. However, coronary vasospasm can trigger coronary thrombosis, and may play an important role in the pathogenesis of acute coronary syndromes, though the precise morphological mechanisms underlying this process remain unclear.

**Case presentation:**

A 43-year-old man with a history of multivessel coronary vasospastic angina had been treated with long-acting diltiazem and fluvastatin since 2004. Eleven years later, following 1 month of medication nonadherence, he experienced recurrence of rest angina and myocardial infarction, with elevated high-sensitivity troponin T. An emergency coronary angiogram demonstrated no de novo lesions, and the current episode was diagnosed as intractable sustained coronary spasm-induced anterior myocardial infarction. Optical coherence tomography imaging revealed the coronary plaque with homogeneous high-intensity signal, and a clearly visualized intraplaque neovascular microchannel (NVMC) network.

**Conclusions:**

Neovascularization within a coronary atheroma is known to accelerate coronary atherosclerosis. The current case with coronary vasospastic angina highlights the role of NVMC formation in this process.

**Electronic supplementary material:**

The online version of this article (doi:10.1186/s12872-016-0408-y) contains supplementary material, which is available to authorized users.

## Background

Coronary plaques in patients with vasospastic angina have been characterized by diffuse intima-media thickening with homogeneous fibrous tissue, without confluent necrotic tissue [1]. However, coronary vasospasm can trigger coronary thrombosis, and may play an important role in the pathogenesis of acute coronary syndromes [2], though the precise morphological mechanisms underlying this process remain under investigation. Neovascularization within coronary atheromas has been known to accelerate coronary atherosclerosis via various mechanisms, including transportation of nourishment to the intima, stimulation of vascular inflammation, and microvascular hemorrhage or leakage. Optical coherence tomography (OCT) has emerged as the most accurate instrument for intracoronary evaluation of plaque features and post-stent vascular responses [3, 4]. Also, OCT imaging can provide cross-sectional in vivo images of neovascular microchannel (NVMC) formation at micrometer resolution. Here, we report an interesting case of NVMC formation at the site of a repeatedly provoked coronary vasospasm, in which intravascular OCT suggested the possible involvement of vasospasm in intra-plaque NVMC formation.

## Case presentation

**Fig. 1:**
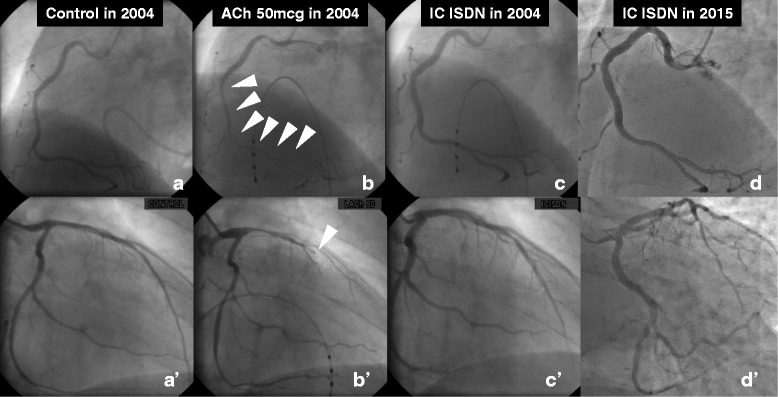
There were no significant obstructions in the right (**a**, **c**) or left (***a***’, ***c***’) coronary artery, but intra-coronary ACh administration induced multivessel spasm (severe vasoconstriction in the distal right coronary artery [arrowheads in **b**]), and complete occlusion of the middle left anterior descending coronary artery [LAD, arrowhead in ***b***’]). After the onset of acute coronary syndrome, an emergency coronary angiogram demonstrated no de novo lesions (Fig. 1**d**, ***d***’)

**Fig. 2:**
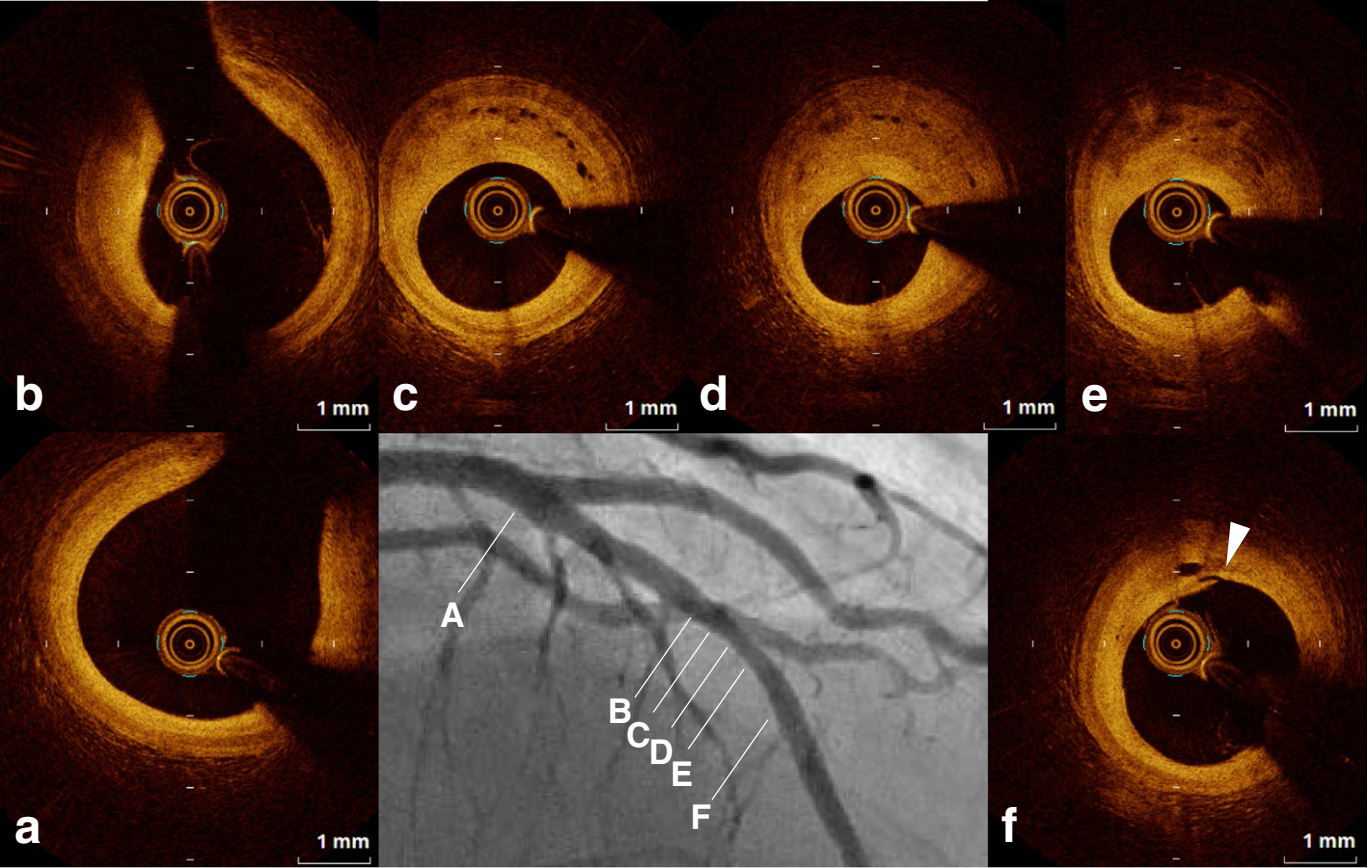
OCT image of the middle left anterior desceinding artery lesion revealed a coronary plaque with homogeneous high signal intensity (Fig. 2**b**, **c**, **d**), and clearly visualized an intraplaque neovascular microchannel (NVMC) network arising from the distal portion (arrowhead, Fig. 2**f**) to the tightest lesion (Fig. 2**d**). The NVMC network spread further proximally, and was distributed within the intra-medial plaque, not connected to the adventitial vasa vasorum (Fig. 2**c**). There were no obstructions at other sites of the LAD (Fig. 2**a**, **b**)

**Fig. 3:**
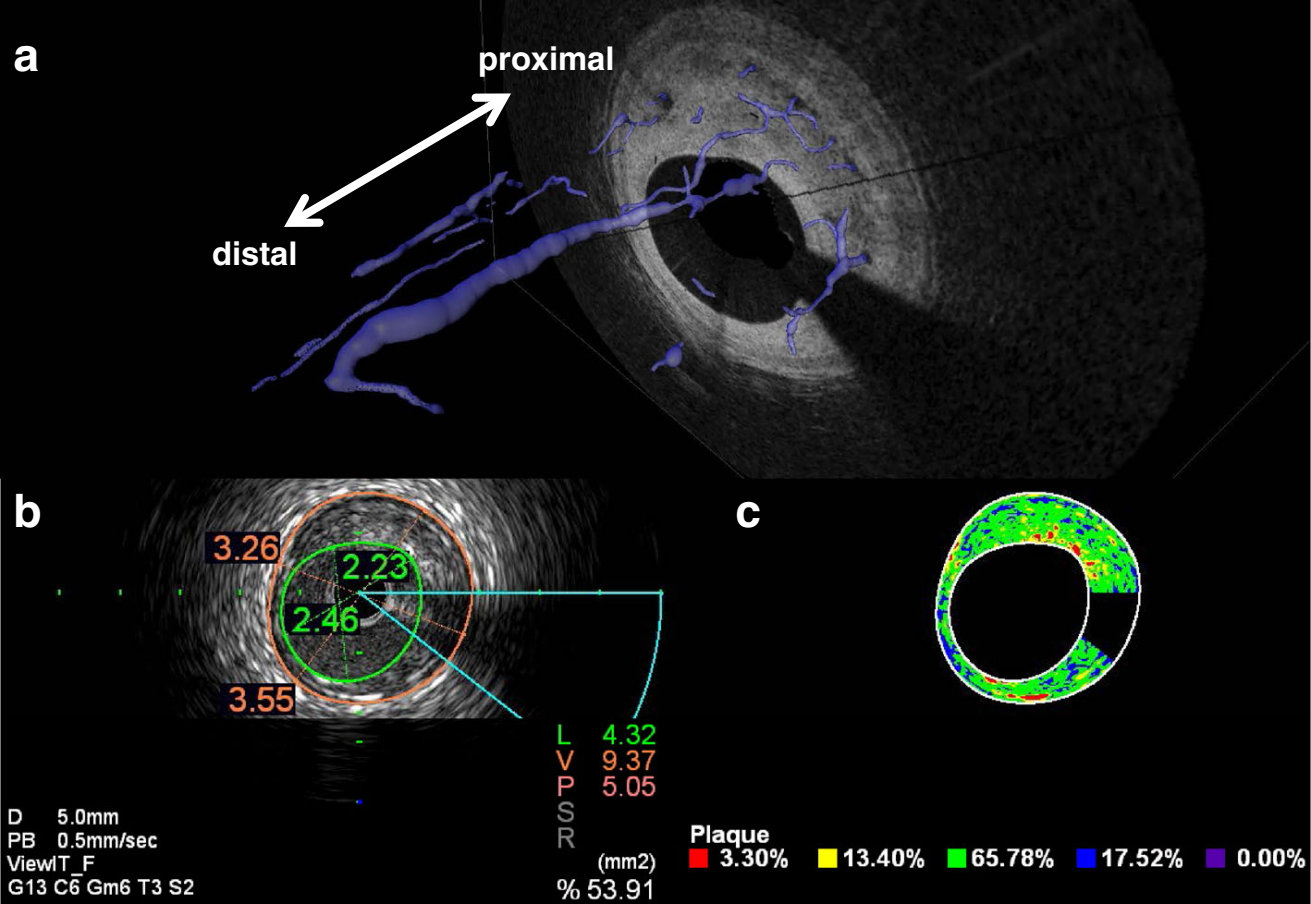
The intra-plaque NVMC was traced automatically using a three-dimensional visualization system, and was reconstructed in three dimensions (Fig. 3**a**). The iso-echoic eccentric plaque on gray-scale intravascular ultrasound (Fig. 3**b**) was mainly composed of fibrous tissue (Fig. 3**c**)


A 43-year-old man with a history of hypertension and sleep apnea syndrome underwent an acetylcholine (ACh) provocation test as part of examination to determine the cause of rest angina in 2004 (Fig. 1a-c and *a’-c’*). There were no significant obstructions in the right (a, c) or left (*a’, c’*) coronary artery, but intra-coronary ACh administration induced multivessel spasm (severe vasoconstriction in the distal right coronary artery [arrowheads in b]), and complete occlusion of the middle left anterior descending coronary artery [LAD, arrowhead in *b*’]). The man was diagnosed with coronary vasospastic angina, and his symptoms completely resolved with anti-angina medications including long-acting diltiazem and fluvastatin. Regarding the moderate stenosis of the middle LAD, revascularization was deferred on the basis of negative findings of exercise stress perfusion imaging. However, rest angina accompanied by myocardial infarction recurred 11even years later, after 1 month of medication nonadherence (maximum high sensitivity troponin T: 1.04 pg/mL). An emergency coronary angiogram demonstrated no de novo lesions (Fig. 1d and *d*’). Intracoronary OCT was performed to provide detailed evaluation of the middle LAD lesions (Fig. 2; Additional file 1). The current episode of cardiac enzyme elevation was diagnosed as intractable sustained coronary spasm-induced anterior myocardial infarction based on the following: 1) absence of plaque rupture or plaque erosion on OCT; 2) abnormal left ventricular anterior wall motion on echocardiography; and 3) clinical history of recurrent rest angina after nonadherence to anti-angina drugs. The OCT image revealed a coronary plaque with homogeneous high signal intensity (Fig. 2b, c and d), and clearly visualized an intraplaque NVMC network arising from the distal portion (direct communication into coronary lumen [arrowhead], Fig. 2f) to the tightest lesion (Fig. 2d). The NVMC network spread further proximally, and was distributed within the intra-medial plaque, not connected to the adventitial vasa vasorum (Fig. 2c). There were no obstructions at other sites of the LAD (Fig. 2a and b). The intra-plaque NVMC was traced automatically using a three-dimensional visualization system (Amira 5.4, Maxnet Co., Ltd., Tokyo, Japan), and was reconstructed in three dimensions (Fig. 3a). The plaque characteristics were assessed by integrated backscatter intravascular ultrasound (ViewIT, Terumo Co., Tokyo, Japan). The iso-echoic eccentric plaque on gray-scale intravascular ultrasound (Fig. 3b) was mainly composed of fibrous tissue (Fig. 3c), as previously reported by our department [1].

Intracoronary optical coherence tomography imaging of intraplaque neovascular microchannel formation at site of coronary spasm.


## Conclusions

Cardiologists have recently become aware that the process of atherosclerotic development is not confined to the intima, but may also be affected by dynamic interactions with the surrounding environment (e.g. vasa vasorum, pericardial fat). Taruya et al. reported that increased intraplaque neovessels were associated with coronary plaque vulnerability [5], suggesting possible relationship between coronary vasospasm and accelerated plaque instability in the current case. Previous studies have shown that diabetes promotes microangiopathic neovessels in the plaque, as well as in the retina [6], while statins prevented neovascularization in hypercholesterolemic pigs, independently of their cholesterol-lowering effect [7]. In the current case, 1 month of nonadherence to anti-angina medication including diltiazem and fluvastatin might have reactivated the patient’s coronary vasospasm, thus promoting rapid development of intraplaque neovascularization.

## Abbreviations

ACh: Acetylcholine
LAD: Left anterior descending coronary artery
NVMC: Neovascular microchannel
OCT: Optical coherence tomography
